# L. C. Dobyns

**Published:** 1869-02

**Authors:** 


L. C. DOBYNS.—Among our advertisements may be seen a notice of the Northwestern Life Insurance Company, of Milwaukee. This company is earning a very favorable reputation in our State. Its officers are gentlemen of undoubted financial ability, and its paid up capital sufficient to meet, promptly, all policies when called for. Our fellow-townsman, L. C. Dobyns, Esq., is the Agent for Lee, Van Buren and Davis counties, and we most heartily recommend him to the favorable consideration of all persons wishing life policies.

